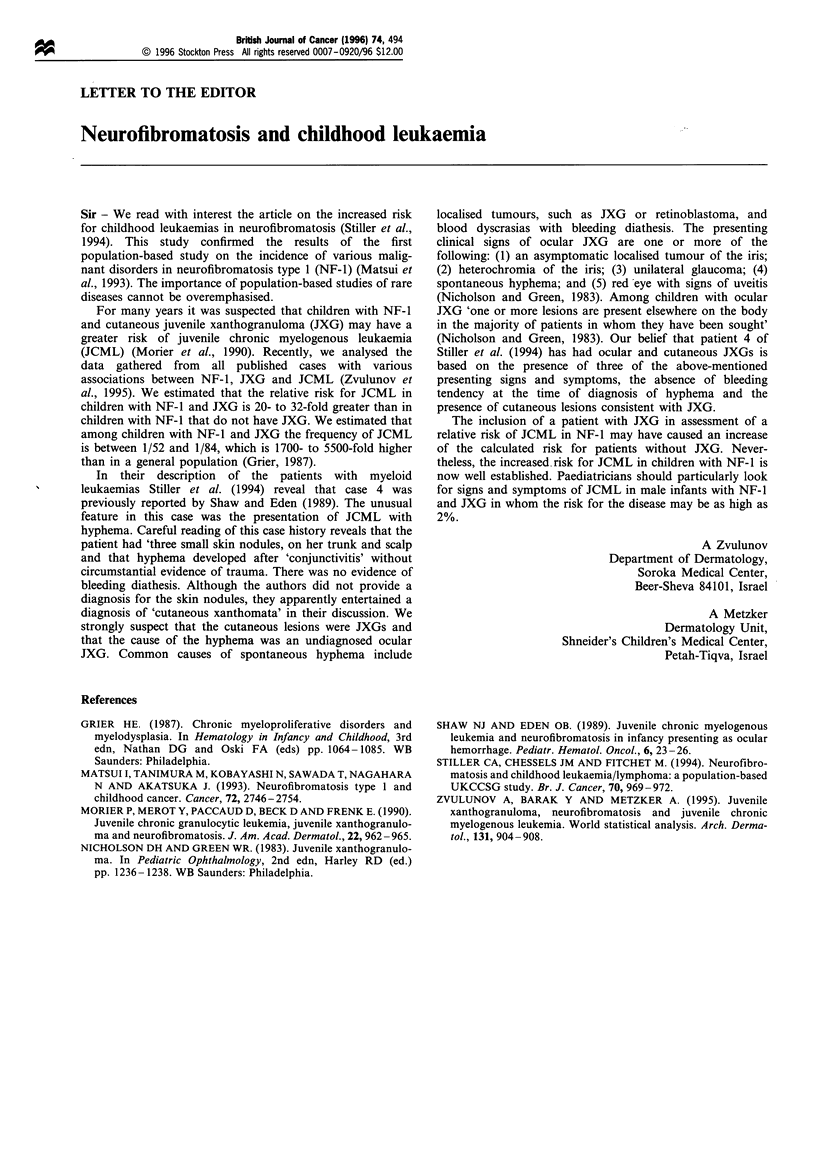# Neurofibromatosis and childhood leukaemia.

**DOI:** 10.1038/bjc.1996.390

**Published:** 1996-08

**Authors:** A. Zvulunov, A. Metzker


British Journal of Cancer (1996) 74, 494
© 1996 Stockton Press All rights reserved 0007-0920/96 $12.00

### LETTER TO THE EDITOR

### Neurofibromatosis and childhood leukaemia

**Sir** – We read with interest the article on the increased risk for childhood leukaemias in neurofibromatosis (Stiller *et al.*, 1994). This study confirmed the results of the first population-based study on the incidence of various malignant disorders in neurofibromatosis type 1 (NF-1) (Matsui *et al.*, 1993). The importance of population-based studies of rare diseases cannot be overemphasised.

For many years it was suspected that children with NF-1 and cutaneous juvenile xanthogranuloma (JXG) may have a greater risk of juvenile chronic myelogenous leukaemia (JCML) (Morier *et al.*, 1990). Recently, we analysed the data gathered from all published cases with various associations between NF-1, JXG and JCML (Zvulunov *et al.*, 1995). We estimated that the relative risk for JCML in children with NF-1 and JXG is 20- to 32-fold greater than in children with NF-1 that do not have JXG. We estimated that among children with NF-1 and JXG the frequency of JCML is between 1/52 and 1/84, which is 1700- to 5500-fold higher than in a general population (Grier, 1987).

In their description of the patients with myeloid leukaemias Stiller *et al.* (1994) reveal that case 4 was previously reported by Shaw and Eden (1989). The unusual feature in this case was the presentation of JCML with hyphema. Careful reading of this case history reveals that the patient had 'three small skin nodules, on her trunk and scalp and that hyphema developed after 'conjunctivitis' without circumstantial evidence of trauma. There was no evidence of bleeding diathesis. Although the authors did not provide a diagnosis for the skin nodules, they apparently entertained a diagnosis of 'cutaneous xanthomata' in their discussion. We strongly suspect that the cutaneous lesions were JXGs and that the cause of the hyphema was an undiagnosed ocular JXG. Common causes of spontaneous hyphema include localised tumours, such as JXG or retinoblastoma, and blood dyscrasias with bleeding diathesis. The presenting clinical signs of ocular JXG are one or more of the following: (1) an asymptomatic localised tumour of the iris; (2) heterochromia of the iris; (3) unilateral glaucoma; (4) spontaneous hyphema; and (5) red eye with signs of uveitis (Nicholson and Green, 1983). Among children with ocular JXG 'one or more lesions are present elsewhere on the body in the majority of patients in whom they have been sought' (Nicholson and Green, 1983). Our belief that patient 4 of Stiller *et al.* (1994) has had ocular and cutaneous JXGs is based on the presence of three of the above-mentioned presenting signs and symptoms, the absence of bleeding tendency at the time of diagnosis of hyphema and the presence of cutaneous lesions consistent with JXG.

The inclusion of a patient with JXG in assessment of a relative risk of JCML in NF-1 may have caused an increase of the calculated risk for patients without JXG. Nevertheless, the increased risk for JCML in children with NF-1 is now well established. Paediatricians should particularly look for signs and symptoms of JCML in male infants with NF-1 and JXG in whom the risk for the disease may be as high as 2%.

A Zvulunov
Department of Dermatology,
Soroka Medical Center,
Beer-Sheva 84101, Israel

A Metzker
Dermatology Unit,
Shneider's Children's Medical Center,
Petah-Tiqva, Israel

### References

GRIER HE. (1987). Chronic myeloproliferative disorders and myelodysplasia. In *Hematology in Infancy and Childhood*, 3rd edn, Nathan DG and Oski FA (eds) pp. 1064–1085. WB Saunders: Philadelphia.

MATSUI I, TANIMURA M, KOBAYASHI N, SAWADA T, NAGAHARA N AND AKATSUKA J. (1993). Neurofibromatosis type 1 and childhood cancer. *Cancer*, **72**, 2746–2754.

MORIER P, MEROT Y, PACCAUD D, BECK D AND FRENK E. (1990). Juvenile chronic granulocytic leukemia, juvenile xanthogranuloma and neurofibromatosis. *J. Am. Acad. Dermatol.*, **22**, 962–965.

NICHOLSON DH AND GREEN WR. (1983). Juvenile xanthogranuloma. In *Pediatric Ophthalmology*, 2nd edn, Harley RD (ed.) pp. 1236–1238. WB Saunders: Philadelphia.

SHAW NJ AND EDEN OB. (1989). Juvenile chronic myelogenous leukemia and neurofibromatosis in infancy presenting as ocular hemorrhage. *Pediatr. Hematol. Oncol.*, **6**, 23–26.

STILLER CA, CHESSELS JM AND FITCHET M. (1994). Neurofibromatosis and childhood leukaemia/lymphoma: a population-based UKCCSG study. *Br. J. Cancer*, **70**, 969–972.

ZVULUNOV A, BARAK Y AND METZKER A. (1995). Juvenile xanthogranuloma, neurofibromatosis and juvenile chronic myelogenous leukemia. World statistical analysis. *Arch. Dermatol.*, **131**, 904–908.